# Role of Cel5H protein surface amino acids in binding with clay minerals and measurements of its forces

**DOI:** 10.1186/s42649-021-00066-7

**Published:** 2021-11-11

**Authors:** Renukaradhya K. Math, Nagakumar Bharatham, Palaksha K. Javaregowda, Han Dae Yun

**Affiliations:** 1 SDM Research Institute for Biomedical Sciences, 5th Floor, Manjushree Building, SDM College of Medical Sciences & Hospital Campus, Shri Dharmasthala Manjunatheshwara University, Dharwad, Sattur 580009 India; 2 Division of Applied Life Sciences, Gyeongsang National University, Chinju, 660701 Republic of Korea; 3 The University of Trans-Disciplinary Health Sciences and Technology (TDU), Bengaluru, Karnataka 560064 India

**Keywords:** Clay mineral, Protein binding, Homology modeling, Mutation, AFM, Adhesion force

## Abstract

Our previous study on the binding activity between Cel5H and clay minerals showed highest binding efficiency among other cellulase enzymes cloned. Here, based on previous studies, we hypothesized that the positive amino acids on the surface of Cel5H protein may play an important role in binding to clay surfaces. To examine this, protein sequences of *Bacillus licheniformis* Cel5H (*Bl*Cel5H) and *Paenibacillus polymyxa* Cel5A (*Pp*Cel5A) were analyzed and then selected amino acids were mutated. These mutated proteins were investigated for binding activity and force measurement via atomic force microscopy (AFM). A total of seven amino acids which are only present in *Bl*Cel5H but not in *Pp*Cel5A were selected for mutational studies and the positive residues which are present in both were omitted. Of the seven selected surface lysine residues, only three mutants K196A(M2), K54A(M3) and K157T(M4) showed 12%, 7% and 8% less clay mineral binding ability, respectively compared with wild-type. The probable reason why other mutants did not show altered binding efficiency might be due to relative location of amino acids on the protein surface. Meanwhile, measurement of adhesion forces on mica sheets showed a well-defined maximum at 69 ± 19 pN for wild-type, 58 ± 19 pN for M2, 53 ± 19 pN for M3, and 49 ± 19 pN for M4 proteins. Hence, our results demonstrated that relative location of surface amino acids of Cel5H protein especially positive charged amino acids are important in the process of clay mineral-protein binding interaction through electrostatic exchange of charges.

**Supplementary Information:**

The online version contains supplementary material available at 10.1186/s42649-021-00066-7.

## Introduction

Several explanations have been provided on the mechanism of protein adsorption on clay mineral surfaces, which states that - electrostatic interactions occur between proteins and surfaces. In particular, the protein becomes more positively charged as the pH decreases below the isoelectric point (p*I*) and the clay remains negatively charged (Burns and Dick 2002; Math et al. 2020). To date, our understanding of the clay minerals-protein interactions and their forces on clay surfaces is still relatively limited. However, some of the previous studies have demonstrated that protein p*I* plays an important role in binding to clay particles in soil and on clay minerals (Burns and Dick 2002; Math et al. 2020). A previous study suggested that ion exchange occurs with the terminal NH^3+^ group that anchors the polypeptide, and the remainder of the polypeptide chain of the enzyme surface amino acids is attracted to the clay surface by van der Waals forces. This implies that these could be the first amino acids formed during the period of evolution Ponnamperuma et al. (1982). Another study suggested that proteins might interact with binding forces such as electrostatic forces through positively charged surface amino acids of the protein hydrolytic domain, laying the road for development of enzymes for ecological applications (Staunton and Quiquampoix 1994; Math et al. 2019). However, none of these studies provided visualization and force measurement data. Studies involving AFM analysis might expand the horizons understanding on clay mineral-protein complexes and forces involved during the process (Zhai et al. 2019).

The phyllosilicates, or sheet silicates, are the vital group of minerals that includes the micas, chlorite, clay minerals etc., and because of their physico-chemical surface properties and other characteristics like surface charge etc., they are proved to be important substrate for various industrial application (Moro et al. 2016). In addition, they are also used as an effective substrate for decontamination of cationic and anionic pollutants from soil, water and organic pollutants like antibiotics (Heinz 2012; Weng et al. 2018). Also, phyllosilicates have capacity for organisation and condensation of biomolecules and cells (Pietrement et al. 2018; Tavanaee et al. 2017).

Clay minerals found in earth crust such as illites (mica and types), smectites (montmorillonite) and kaolonites are chemically active in the environment (Weaver and Pollard 1973). Illites and smectites are 2:1 layer silicates (two Si-O sheet in each layer) and kaolinite are 1:1 layer silicates (one Si-O sheet in each layer) (Kloprogge et al. 1999). Previous studies on absorption of biological molecules like amino acids involving kaolinite and montmorillonite clay minerals provided information that aa interact with the clay surfaces via of exchangeable metal cation present on the high surface area of minerals (Porter et al. 2000). Besides, clay minerals are aluminosilicates and possess acidic sites that are capable of interaction with aa of protein via ionic binding (Sanjay & Sugunan 2005). Also, presence of edges and cracks on the surface of minerals are crucial for reactions to occur. On the same lines, our previous study also observed high amount of proteins on the edges of clay minerals and some absorbed in the cracks upon observing images taken from Atomic Force Microscopy (Math et al. 2019). AFM, a powerful tool used for single-molecule interaction between protein-ligand (Sumbul and Rico 2019), clay minerals-protein (Math et al. 2019), affinity under in-vitro conditions and protein-substrates like cellulose (Liu et al. 2020). Also, rupture forces of cellulose binding module and fiber substrates of cellulose, were measured using AFM (Griffo et al. 2019), and an adhesive force were successfully measured between pesticide degrading enzyme-soil particles (Islam et al. 2017). These exclusive evidences motivated us to use AFM to understand molecular mechanism and measure the adhesive forces involved between clay minerals-protein in current study. Such studies would help us in developing enzymes for environmental application which can bind to natural soil particles efficiently and stable.

The prediction of structure of biomolecules like protein helps in studying protein-protein, protein-substrate and protein-fiber interactions (Moro et al. 2016; Liu et al. 2020; Moro et al. 2020). The amino acid (aa) sequence of a protein, the so-called primary structure, can be easily determined from the sequence on the gene that codes for it. In the vast majority of cases, this primary structure uniquely determines a structure in its native environment. Knowledge of this structure is vital in understanding the function of the protein. In proteomic, homology modelling is used to predict the structure and function of a protein/gene: if the sequence of gene, whose function is known, is homologous to the sequence of gene*,* whose function is unknown, one could infer that both the proteins may share the same function. In the structural branch of bioinformatics, homology is used to determine which parts of a protein are important in structure formation and interaction with other proteins and sometimes with negatively charged clay minerals. This method currently remains the only way to predict protein structures reliably (Barnes and Gray 2003) which could be useful in predicting proteins which interact with clay particles.

Previous studies affirmed through FTIR analysis demonstrated that α-chymotrypsin adsorption of montmorillonite involves electrostatic exchange from the electronegative charged clay surface and positively charged protein side chain surface amino acids such histidine, lysine, arginine (positively charged aa). They also confirmed that binding of protein with electrostatic interaction between these side chains and the clay do not hinder the access of the substrate to the enzymatic site until orientation of the bound enzymes is not appropriate (Baron et al. 1999; Math et al. 2019). However, there are no reports on cloned and expressed proteins and measurements of such forces involved in binding with clay minerals.

In the present study as a first step we aim; to predict and identify surface amino acids in Cel5H protein which interact with clay minerals by performing mutational analysis using bioinformatics tools. As a second step, to clone and investigate the mutated Cel5H proteins on clay minerals, and lastly, to measure the forces involved during protein and mica surface interaction using AFM. Further, we intend to propose a model to explain clay minerals-protein- binding interaction.

## Methods

### Sequence alignment and site directed mutation of Cel5H surface amino acids

**Fig. 1:**
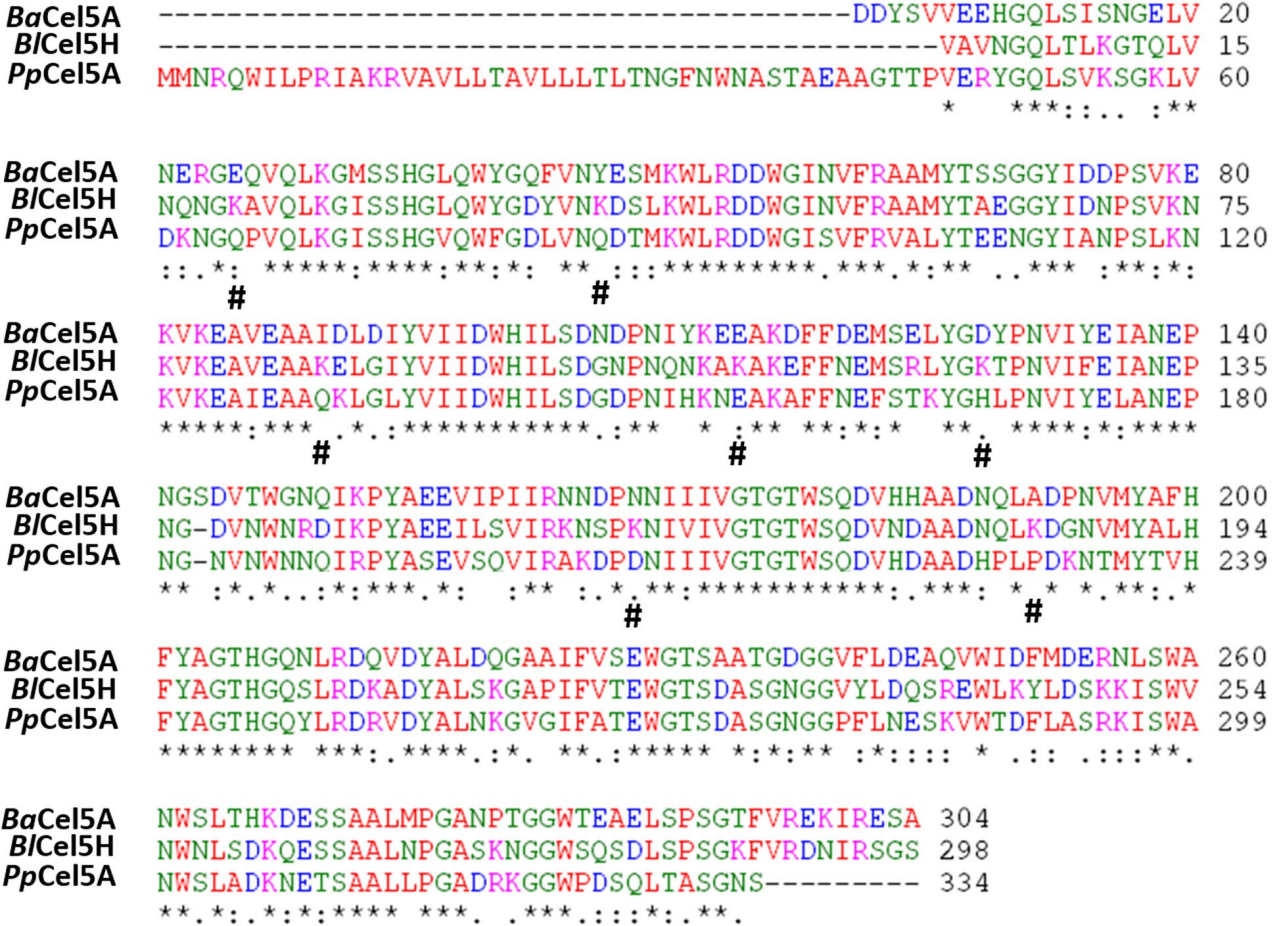
Sequence alignment of *Pp*Cel5A *and Bl*Cel5H proteins. Identical residues are represented with asterisk (*) and specific charged residues in *Bl*Cel5H which are selected for mutational studies are highlighted with #. *Ba: Bacillus agaradhaerens, Bl: Bacillus licheniformis* and *Pp* represents *Paenibacillus polymyxa*

Sequence alignment was performed by ClustalW alignment method to analyze and compare the *Bl*Cel5H and *Pp*Cel5A sequences. Based on the conserved amino acids (aa’s) and position of aa on the glycoside hydrolase (GH) domain of Cel5H protein sequence, seven aa’s were selected for mutation study (Fig. 1).

The oligonucleotide primers designed by overlapping complementary primers containing the desired nucleotide changes were designated for each mutation as mentioned in Table 1. Single mutant plasmid DNA was used as template for double mutation. The procedure for the site directed mutagenesis was conducted according to the manufacturer’s specifications (Site-directed mutagenesis kit, Stratagene, La Jolla, CA). Site-directed mutagenesis of the plasmid harboring *cel*5H gene to create K219A(M1), K196A(M2), K54A(M3), K157T(M4), K75A(M5), K119A(M6), and K143A(M7) mutations for putative binding sites in Cel5H (Cho et al. 2008).

**Table 1:** Nucleotide sequence of used oligonucleotide primer

Mutation	Sequence (5′➔3′)	Mutant amino acid
K219A(M1)	^a^F-GAAATTGCCAAC**GCT**CCGAACGGCGAT(AAA ➔ GAA) R-AATCGCCG**AGC**GGTTCGTTGGCAATTT	Lys ➔ Ala
K196A(M2)	F-AAAAACTCTCCG**GCT**AATATTGTGATT (AAA ➔ GAA) R-ATCACAATATT**AGC**CGGAGAGTTTTTG	Lys ➔ Ala
K54A(M3)	F-GTCAATCAAAACGGA**GCT**GCGGTTCAGC (AAA ➔ GAA) R-AGCTGAACCGC**AGC**TCCGTTTTGATTGA	Lys ➔ Ala
K157T(M4)	F-TCAAGACTTTATGGC**ACG**ACGCCAAACG (AAG ➔ ACG) R-ACGTTTGGCGT**CGT**GCCATAAAGTCTTG	Lys ➔ Thr
K75A(M5)	F-CCGTCGAAGCGGCA**GCT**GAACTCGGAATCT (AAA ➔ GAA) R-GATTCCGAGTTC**AGC**TGCCGCTTCGACGGC	Lys ➔ Ala
K119A(M6)	F-ACCAAAACAAAGCG**GCT**GCAAAAGAATTTTT (AAA ➔ GAA) R-AAAAATTCTTTTGC**AGC**CGCTTTGTTTTGGT	Lys ➔ Ala
K143A(M7)	F-CGATTATGTCAAC**GCT**GACTCGTTAAAAT (AAA ➔ GAA) R-TTTTAACGAGGC**AGC**GTTGACATAATCGC	Lys ➔ Ala

^a^Mismatches with the original sequence of the *cel*5H gene are underlined

### Homology Modelling and molecular docking simulations of the *Bl*Cel5H and *Pp*Cel5A

Meanwhile, as we do not have crystal structure information of *Bacillus licheniformis* Cel5H (*Bl*Cel5H) and *Paenibacillus polymyxa* Cel5A (*Pp*Cel5A), we have adopted computational methodology such as homology modeling to develop three dimensional structures. The coordinates of the crystal structure of *Bacillus agaradhaerens* endoglucanase Cel5A, (PDB ID: 1h5v, 1.10 Å resolution) (Varrot et al. 2001) was used as template to build the initial *Bl*Cel5H and *Pp*Cel5A structures using Modeller program (Sali and Blundell 1993; Fiser et al. 2000; Bharatham et al. 2007).

The binding interactions can be ascertained by docking the substrates/inhibitors into the active site of the protein. The GOLD 3.1 (Jones et al. 1997; Bharatham et al. 2008) program was used to calculate the docking modes of cellulose into the active sites of the homology modeled protein structures (CBM domains of *Bl*Cel5H and *Pp*Cel5A). GOLD considers complete ligand flexibility and partial protein flexibility and the energy functions are partly based on conformational and non-bonded interactions. The following default genetic algorithm parameters were used: 100 population size 1.1 for selection, 5 number of islands, 100,000 number of genetic operations and 2 for the niche size.

### Expression and purification of Cel5H and mutant proteins

For high expression of Cel5H mutants, the PCR product generated with primers were cloned into the expression vector pET-28a(+) using *Nde*l and *Sac*l sites, resulting in addition of a N-terminal thrombin tag as mentioned previously (Math et al. 2019). Purification of protein with twenty-two amino acid residues including a His-Tag and a thrombin cleavage site fused in frame to the N-terminal end of Cel5H was done as previously described (Guo et al. 2005).

### Tryptophan emission assay of mutant enzymes to assess structural changes

Tryptophan emission fluorescence spectra of Cel5H and its mutant proteins were measured on a LS-45 fluorescence spectrometer (PerkinElmer, USA) at an excitation wavelength of 290 nm using cuvettes with an optical path length of 1 cm. The emission spectra of protein samples with a concentration of 0.20 μM in 20 mM TrisHCL buffer (pH 7.0) were measured from 300 to 400 nm (excitation and emission slit width = 5 nm) under the scanning speed of 240 nm/min. The temperature was maintained at 37 °C using an external bath circulator. All fluorescence spectra were corrected for background scattering with pure buffer.

### Binding assay of mutant proteins to analysis binding pattern

The binding assay of wild-type and mutant proteins on clay minerals was carried out as mentioned previously by Math et al. (2019). In brief, clay mineral suspensions were prepared in water and pH was adjusted to 7. The complex suspensions were agitated (KSI-100 L shaking incubator) at 25 °C until equilibrium was reached. Protein present was estimated using Bio-Rad protein assay kit (Bio-Rad Laboratories, Hercules, CA, USA). The amount of enzymes bound was estimated using the formula as previously described (Safari Sinegani et al. 2005; Math et al. 2019).

### Force spectroscopy analysis of wild-type and mutant Cel5H enzymes on mica

For the protein–clay mineral complexes, after the equilibrium was reached, as mentioned previously by Math et al. (2020) the suspensions were centrifuged, resuspended in 5 ml of deionized distilled water and sonicated in an ice bath for 2 min at 140 W. A few drops of the protein sample were deposited on a mica sheets. The mica sheet was fastened to a magnetized stainless steel disk with double-sided tape. The AFM three-dimensional images of the samples were taken, under similar conditions, with a XE-series atomic force microscope (Park Systems corp. Suwon, Korea) in air and at room temperature (25 °C). The measurements were performed in AFM contact mode with 910 M-NSC36 (official name NSC36/ALBS) a silicon cantilever with a spring constant of 0.6 N/m was used and the scanning frequency was 22.0 Hz. As mentioned in the above paragraph purified Cel5H proteins was spread on mica sheets and imaged by contact mode as explained (Wang et al. 2002; Wright and Revenko 2004). Adhesion or pull off forces for wild and mutant Cel5H proteins were measured by following as in approach curve acquisition for XE-series SPM operation manual (Park systems corporation, Suwon, Korea) Cantilever spring constants were calibrated using a reference cantilever of a precisely controlled force constant (Tortonese and Kirk 1997). Five hundred retraction force curves recorded on arbitrary different locations are summarized in the form of histogram and represented as a probability (%) (Okada et al. 2008).

## Results

### Sequence alignment and site directed mutation of Cel5H surface amino acids

On comparing sequences of *Bl*Cel5H and *Pp*Cel5A, we observed remarkable presence of positively charged residues (lysine/arginines) specifically in *Bl*Cel5H. We then selected only the positive residues which are specifically present in *Bl*Cel5H (Fig. 1) for further mutational studies. Positive residues which were present in both the proteins were omitted. Therefore, it is assumed that if the protein is to bind on clay mineral surface, only the hydrolytic domain (GH) might be responsible for interacting with the clay surface rather than the substrate binding region (Math et al. 2020). Hence, positive surface amino acids are presumed to play an important role in binding proteins on clay surfaces.

### Homology Modelling and molecular docking simulations of the *Bl*Cel5H and *Pp*Cel5A

**Fig. 2:**
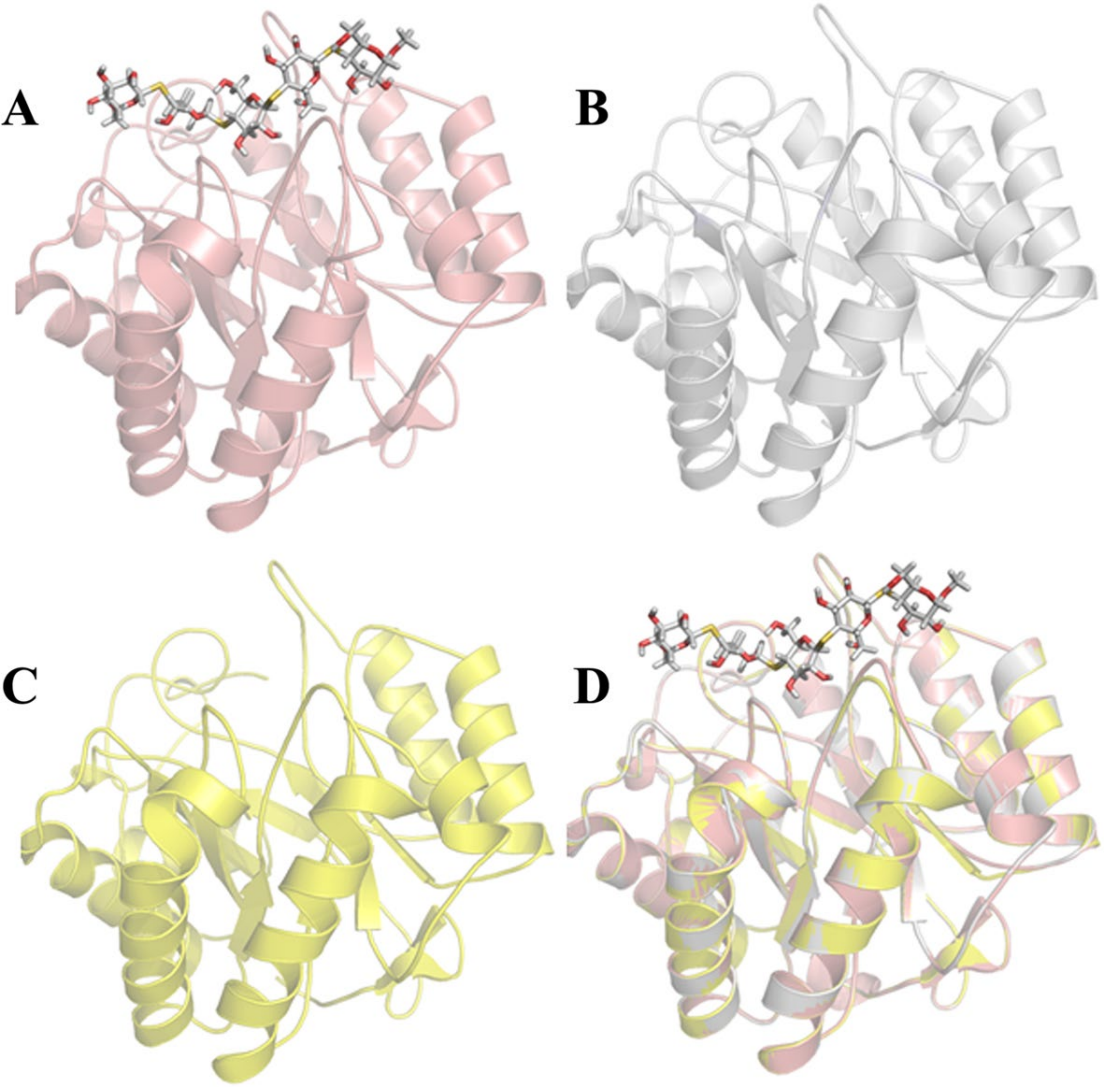
Ribbon diagrams of *Ba*Cel5A crystal structure (PDB ID: 1H5V) **A**. *Bl*Cel5H homology model **B**, *Pp*Cel5A homology model **C**, and superimposition of template and model structures **D**. The cellulose mimic (4,4^II^,4^III^,4^IV^-tetrathio-α-cellopentoside) which is in bound form with *Ba*Cel5A was represented with sticks

Molecular docking studies performed to understand the cellulose binding pattern and important residues that can interact with cellulose. The crystal structure of *B. agaradhaerens* endoglucanase (Cel5A) was comprised with cellulose mimic (4,4II,4 III,4 IV -tetrathio-cellopentoside). We have taken the ligand information to detect the cellulose binding site in homology modeled structures by aligning with crystal structure. We have also taken advantage from this ligand information to predict the binding cavity atoms for GOLD docking studies (Fig. 2). Above analysis provided probable information of cellulose and protein clay mineral interaction sites on Cel5H. However, later was confirmed by experimental analysis.

### Tryptophan emission assay of mutant enzymes to assess structural changes

To evaluate possible structural changes that may result from mutations, wild-type Cel5H and its mutant forms were compared using fluorescence spectra. The wavelength of the emission maximum (*λ* max) for tryptophan (Trp) depends on its microenvironment. Specifically, a low polarity, hydrophobic microenvironment is characterized by *λ*max ˜ 331 nm, while for Trp in an aqueous phase is 350–353 nm (Burstein et al. 1973). The tryptophan emission fluorescence spectrum for wild-type Cel5H was maximized at a wavelength 339.5 nm (supplementary Fig. 1). Meanwhile, the spectra of mutant protein M1, M2, M3, M4, M5, M6 and M7 were not redshifted with a peak centred at 339.1, 339.1, 339.2, 339.3, 339.2 and 339.1, respectively indicating the environment of tryptophan residues was not altered in these mutant proteins (data not shown).

### Binding assay of mutant proteins to analysis binding pattern

The mutant proteins expressed were subjected to enzymatic activity and all showed no change in cellulase activity. Further, purified mutant proteins’ binding test revealed that among seven mutants tested only three mutant proteins M2, M3, M4 showed changes in their binding activity, specifically reduced binding capacity by 12%, 7% and 8%, respectively (Table 2).

**Table 2:** Total binding of wild-type mutant Cel5H protein on clay minerals

Cel5H mutant	Mutant protein binding ability (%)^**a**^	Change in enzyme activity after mutation (U mg^**− 1**^) pH 7.0
K219A(M1)	99.2	424.10
K196A(M2)	87.81 (12% decrease)	424.11
K54A(M3)	92.88 (7% decrease)	423.90
K157T(M4)	91.91 (08% decrease)	424.00
K75A(M5)	99.1	424.00
K119A(M6)	99.3	424.03
K143A(M7)	99.3	424.00

^a^change in binding capacity [compared to wild-type (99.5%)] is calculated as mentioned in our previous article (Math et al. 2019)

### Force spectroscopy analysis of wild-type and mutant Cel5H protein on mica

**Fig. 3:**
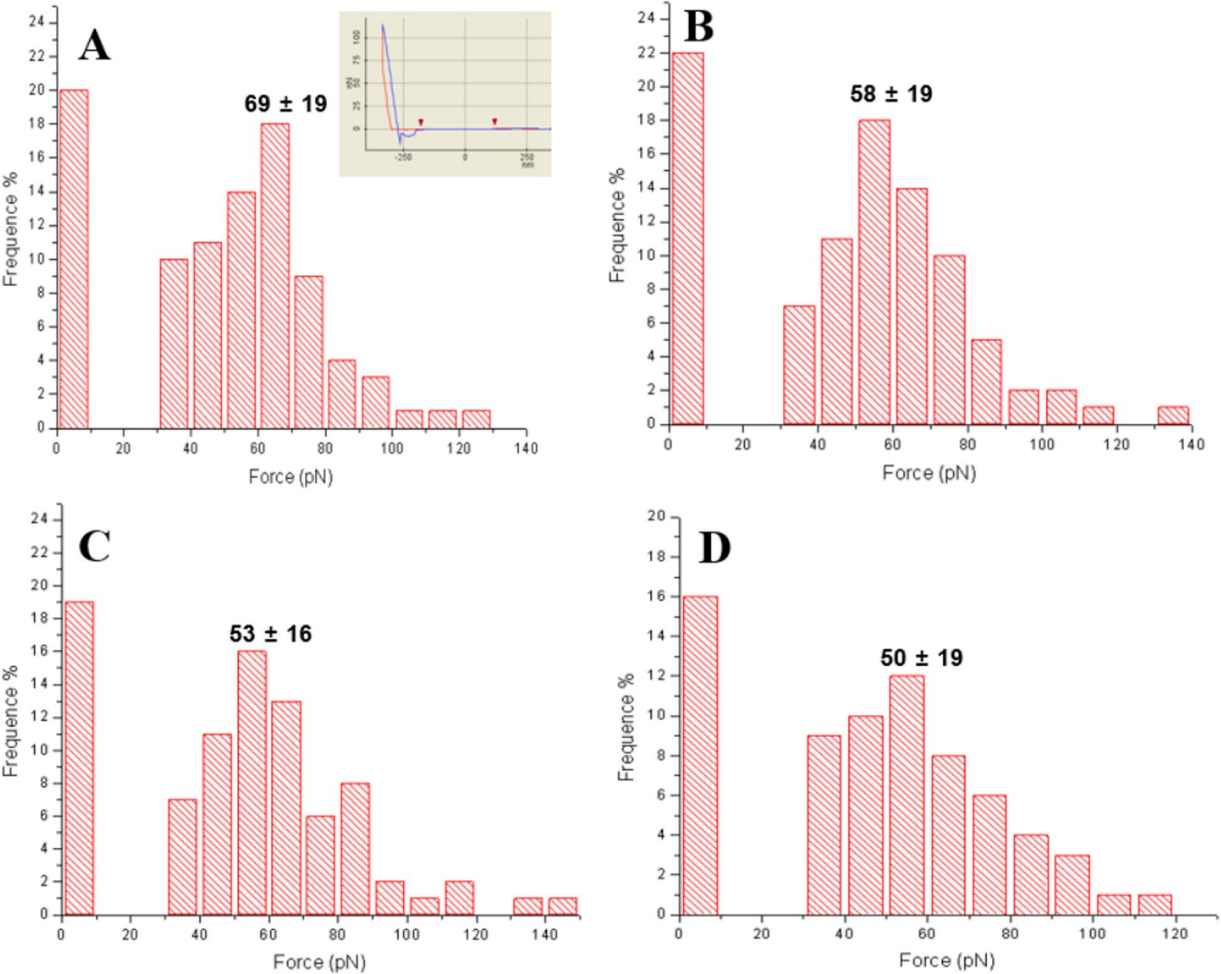
Measurement of adhesion force using AFM of wild and mutant Cel5H proteins on mica. Adhesion force map for wild-type protein A (Cel5H, gray sclae: 180 pN), adhesion force histogram (*n* = 226), adhesion force histogram for mutant M2 protein B, adhesion force histogram for M3 protein C, adhesion force histogram for M4 protein D. All curves were obtained using an approach and retraction speed of 0.5 μm s^− 1^

To measure the adhesion forces involved between amino acids of wild-type and mutant Cel5H proteins and clay minerals surface, proteins were overlaid on mica to generate the adhesion force histogram and representative nano-force curve for wild-type and mutant Cel5H proteins (pulling velocity of 0.5 μm/s) (Fig. 3). The distribution of the last adhesion forces, occurring typically at 10 ~ 100 nm, showed a well-defined maximum at 69 ± 19 pN for wild-type, 58 ± 19 pN for M2, 53 ± 16 pN for M3, and 50 ± 19 pN for M4 proteins (Fig. 3a-d). The average detachment force and length for all proteins were found in the range of 40 to 70 ± 21 pN and 10 to 100 ± 17 pN, respectively, with the detachment force being lower than the nonspecific interaction at 1 to 10 pN (Radmacher et al. 1994). Meanwhile, adhesion force values on bare mica sheets were between 1 to 2 pN (data not shown). Thus, the pull-off force curves that Cel5H adopts on mica is a relatively compact structure. With pulling off the tip, significant adhesion was observed for wild-type protein that gradually decreased for mutants (Fig. 3a-d). A high frequency histogram for zero-force was observed is supposed to be because of friction when tip come into contact with the surface of mica (Fig. 3) after selecting protein, similar pattern was observed in all our analysed samples.

## Discussion

**Fig. 4:**
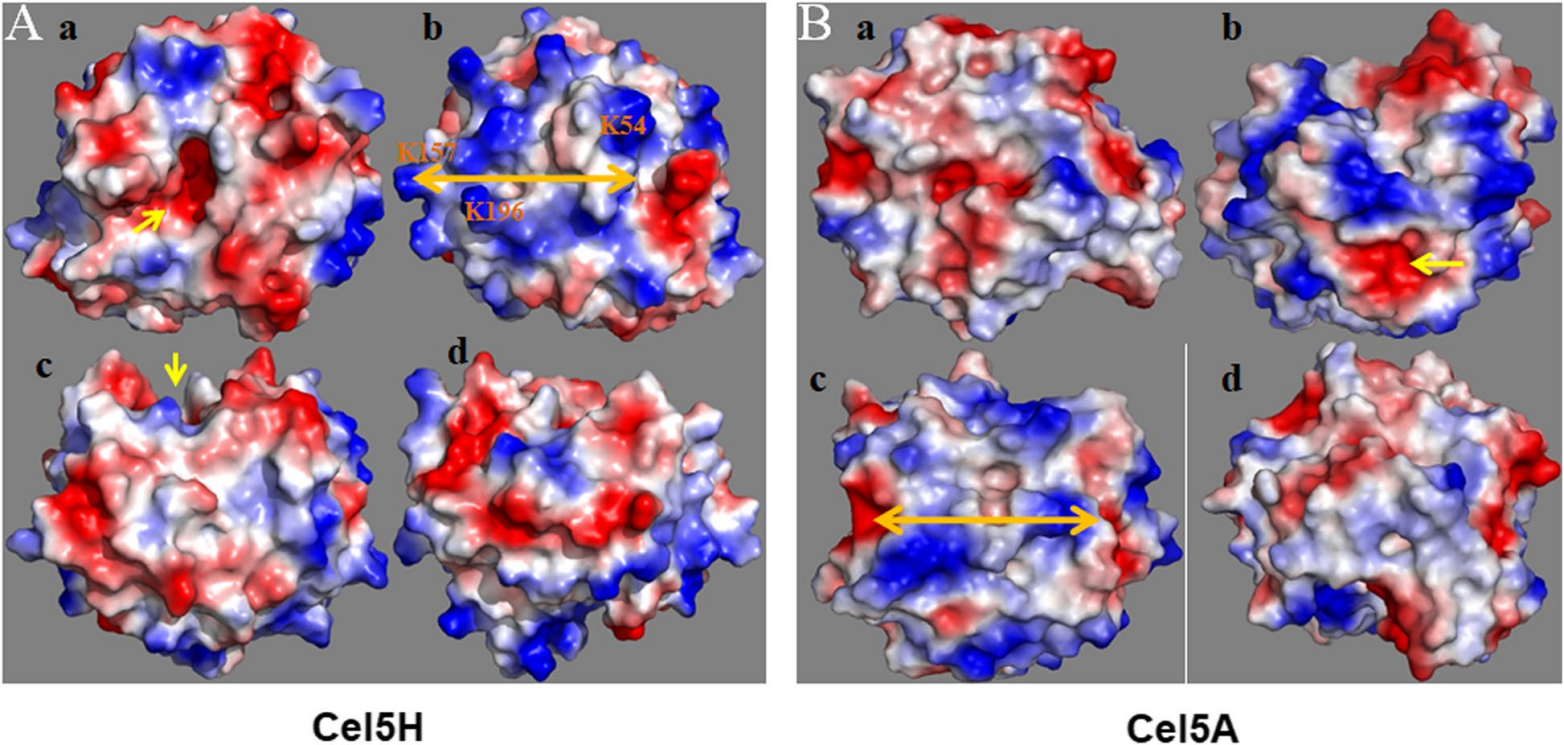
Electrostatic potential energy surfaces of the Cel5H (panel **A**) and Cel5A (panel **B**) proteins. Also, proposed clay mineral and substrate binding sites of the *Bacillus licheniformis* protein (indicated by arrows). Panel **A** & **B**. Views of the front and back side electrostatic surfaces of Cel5H protein (a and b). Views of side electrostatic energy surface of Cel5H protein (c and d), blue is electropositive and red is electronegative. Panel **B**, different views of Cel5A protein, front and back view (a and b), side view (c and d). Substrate binding groove (→) surrounded by electronegative amino acids and clay binding site (↔) filled with electropositive amino acids

Many theories had been proposed stating the importance of protein p*I* in binding to clay surfaces through biochemical assays (Burns and Dick 2002; Math et al. 2019). Our previous study states that only Cel5H protein can bind strongly to clay minerals, while other cloned enzymes bound weakly to clay minerals (Math et al. 2019). They speculate that this could be due to the lack of sufficient total surface charge or total number/location of positively charged amino acids present on the clay mineral binding region of the Cel5H protein. However, in this study our results speculate that only a part of the glycosyl domain of the Cel5H protein participates in binding interactions and the active site of the enzyme is located on the opposite site of the clay mineral binding region (Fig. 4A a,b).

In the present study, though *Bl*Cel5H and *Pp*Cel5A shared around 65% sequence identity, *Pp*Cel5A was unable to demonstrate the clay mineral binding unlike *Bl*Cel5H. Probable reasons for not binding were evaluated by performing the computational and experimental methodologies. At first, we analysed the involvement of cellulose substrate interacting residues by comparing the docked conformations. The structural comparison of 5 Å around the substrate revealed that the key residues are identical in both *Bl*Cel5H and *Pp*Cel5A. So, it was assumed that the positively charged residues which are present in both the proteins at same position may not have the clay mineral binding properties. Then, we have compared other surface residues especially positively charged lysines. Some of these positively charged aa residues in *Bl*Cel5H were replaced with neutral charged aa in *Pp*Cel5A (Fig. 1). This key difference encouraged us to check the hypotheses experimentally by performing the mutation studies in *Bl*Cel5H. Meanwhile, tryptophan emission fluorescence spectrum observations implicated that the significant structural changes have not occurred as a consequence of the mutations. The relative decrease in binding of K196A (M3) could be attributed to change of amino acid into negative/neutral charge which may increase electrostatic repulsion (Staunton and Quiquampoix 1994) or decrease total protein surface charge.

Consequently, adhesion forces were measured on mica sheets as mica hold negative charge (Wright and Revenko 2004; Yin et al. 2012) on its surface which can mimic as clay. Moreover, measuring the adhesion force on clay is quite difficult because of chances of tip damage and more difficult in pulling out proteins from clay surfaces (Zhai et al. 2019). The hydrophilic surface of mica can turn to hydrophobic gradually upon exposure to air. Illite mica does not shrink or swell with drying and wetting unlike kaolinite and monmorillonite. This could be because mica differs from clay minerals by having many fewer random cation substitutions in its crystals because of which mica crystalline sheets are far larger than clay crystals (Sposito et al. 1999). However, large mica sheets exert transducing forces to make covalent bonds with biological molecules like aa. What makes mica more interesting is the amino group (NH_2_) of aa can readily exchange with K+ on the mica sheets. To understand the biological significance of mutant protein binding, it is interesting to measure and compare the adhesion force values here on mica sheets. We observed substantial decreases in total strength of mutant proteins on mica surfaces compared to wild-type, patterns of decreasing adhesion forces (histogram) of mutant proteins through AFM (Fig. 3). Comprehensive results from biochemical assay and adhesion forces reveal that electrostatic, van der waals and chemical forces might be involved in protein binding to clay minerals. Adhesion forces recorded on mica sheets do not exactly imitate the forces on original clay; however, they provide vital information about the nanodomains that may play important functions in clay mineral binding.

The average detachment forces for all proteins were found in range 40-70 ± 21 pN which is lower than nonspecific interaction 1-10 pN (Radmacher et al. 1994). The average detachment length for all proteins was found in range 10-100 ± 17 pN (Mueller et al. 1999). For Cel5H proteins on mica we conclude from the pull-off forces curves that Cel5H adopts a relatively compact structure. Hence, any binding force should be significantly different (Mueller et al. 1999). Pull off the tip adhesion was observed for wild-type protein that gradually decreased in strength for mutant proteins (Fig. 3). Most curves show single pull off forces. The distribution of the last adhesion forces decreased between wild-type and mutant proteins.

Based on our experimental results we suppose that binding of protein with clay minerals like kaolinite and monmorillonite are different from mica surface. Clay minerals might undergo ionic binding whereas mica to make covalent bonds during interaction with aa.

**Fig. 5:**
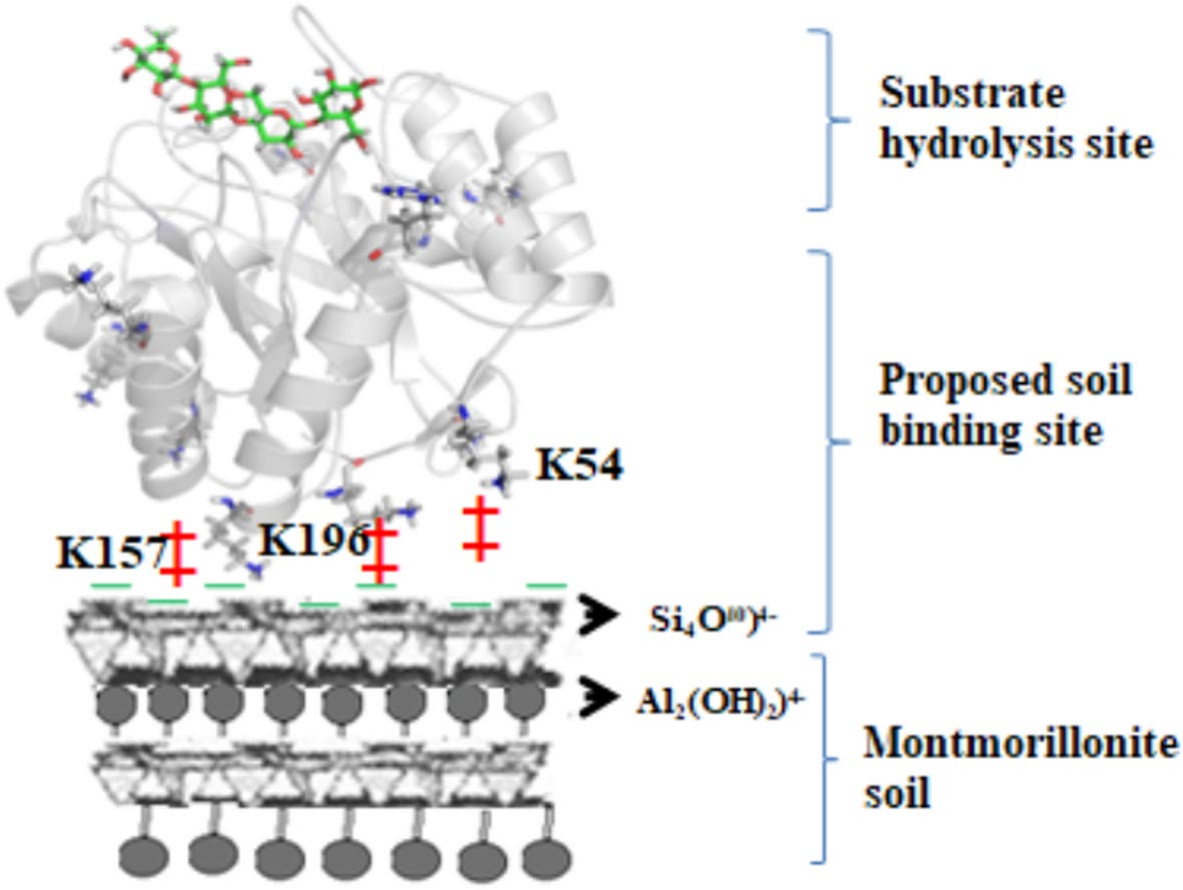
Model of proposed protein-clay mineral-substrate complex, depicting binding of Cel5H protein with surface positive amino acids on clay mineral surface

Based on our results we are proposing a clay mineral-protein binding model (Fig. 5) which explains how negatively charged clay mineral allows positively charged protein domain to bind with electrostatic forces. This model reveals importance of total surface charge of protein and relative location of positively charged aa for efficient binding to clay in natural soil. Understanding of such molecular mechanism helps us in developing enzymes used for bioremediation, pesticide degradation and nutrient mineralization. Also, can be helpful in increasing soil fertility. However, research laboratories and industries should come to together to test such hypothesis for the benefit of farmers and the Earth.

In conclusion, the present study results demonstrate that mutations in Cel5H protein decreased binding ability to clay minerals. The positively charged aa’s present on the surface of the GH domain are involved in binding through clay surfaces, especially, positively charged lysine amino acids might play a key role. Also, we were successful in measuring adhesion forces of Cel5H protein on mica sheets with well-defined maximum.

## Supplementary Information

**Additional file 1.**

## Data Availability

No applicable.
