# Supplementary material for: Role of Cel5H protein surface amino acids in binding with clay minerals and measurements of its forces
Source: Appl Microsc. 2021 Nov 11;51:17. doi: 10.1186/s42649-021-00066-7 (PMC8586110; doi:10.1186/s42649-021-00066-7)
Supplement: Supplementary file 1 — Additional file 1. [file 42649_2021_66_MOESM1_ESM.docx]

**Microscopy**

**Role of Cel5H protein surface amino acids in binding with clay minerals and measurements of its forces**

**Renukaradhya K. Math^1,3*^, Nagakumar Bharatham^2^, Palaksha K. Javaregowda^1^, Han Dae Yun^3^**

^1^SDM Research Institute for Biomedical Sciences, 5^th^ Floor, Manjushree Building, SDM College of Medical Sciences & Hospital campus, Shri Dharmasthala Manjunatheshwara University, Sattur-580009, Dharwad, India

^2^The University of Trans-Disciplinary Health Sciences and Technology (TDU), Bengaluru, Karnataka-560064, India

^3^Division of Applied Life Sciences, Gyeongsang National University, Chinju 660701, Republic of Korea

***Correspondence:** Renukaradhya K. Math

^1^SDM Research Institute for Biomedical Sciences, 5^th^ Floor, Manjushree Building, Shri

Dharmasthala Manjunatheshwara University, Sattur-580009, Dharwad, India

Email: aradhya.swamy@gmail.com

**Supplementary Figure**


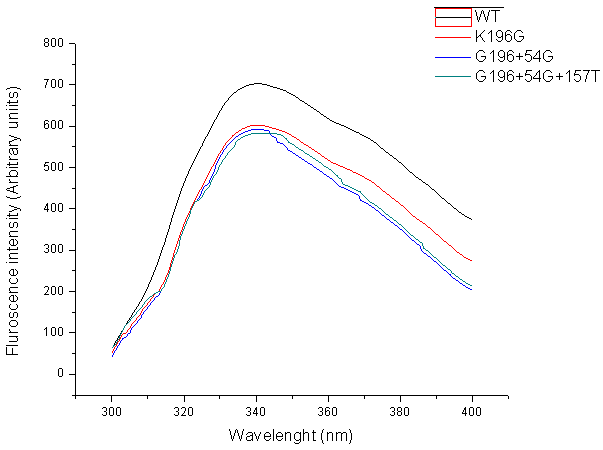

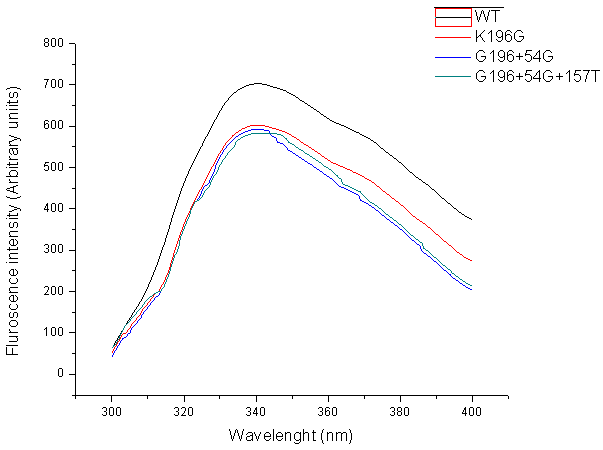


Wild-type

K196A

K54A

K157T

**Supplementary Fig.** Intrinsic tryptophan fluorescence emission spectra of Cel5H and its mutant proteins.
